# Reversal Effects of Royal Jelly and Propolis Against Cadmium-Induced Hepatorenal Toxicity in Rats

**DOI:** 10.1007/s12011-023-03775-0

**Published:** 2023-07-27

**Authors:** Eman M. Omar, Norhan S. El-Sayed, Fatma Y. Elnozahy, Eman Hassan, Alaa Amr, Maria Augustyniak, Lamia M. El-Samad, Abeer El Wakil

**Affiliations:** 1 Department of Medical Physiology, Faculty of Medicine, Alexandria University, Alexandria, 21519 Egypt; 2 Department of Biological and Geological Sciences, Faculty of Education, Alexandria University, Alexandria, 21526 Egypt; 3 Department of Zoology, Faculty of Science, Alexandria University, Alexandria, 21568 Egypt; 4 Institute of Biology, Biotechnology and Environmental Protection, Faculty of Natural Sciences, University of Silesia in Katowice, Bankowa 9, 40-007 Katowice, Poland

**Keywords:** Natural products, Honey bee, Oxidative stress, Toxicity

## Abstract

Heavy metal toxicity is an exponentially growing health problem. In this study, we aimed to assess the protective properties of propolis and royal jelly against cadmium adverse effects. Thirty-two adult male rats were included in our study; kidney and liver functions, histopathological changes, and the level of oxidative stress were evaluated in rats exposed to a daily dose of 4.5 mg cadmium per kilogram of body weight for 1 month and those cotreated simultaneously with either propolis (50 mg/kg/day) or royal jelly (200 mg/kg/day) with cadmium compared to control animals. Cadmium-mediated hepatorenal toxicity was manifested as per the increased oxidative stress, function deterioration, and characteristic histopathological aberrations. The supplementation of royal jelly or propolis restores most of the affected parameters to a level similar to the control group. However, the parameters describing the grade of DNA damage and the interleukin-1β expression in the liver, as well as the levels of malondialdehyde and metallothionein, were slightly elevated compared to controls, despite the regular use of royal jelly or propolis. It is worth noting that better results were found in the case of royal jelly compared to propolis administration. Most likely, the ability of both products to chelate cadmium and contribute in reducing oxidative stress is of great importance. However, further investigations are needed to complement the knowledge about the expected nutritional and medicinal values.

**Graphical abstract:**

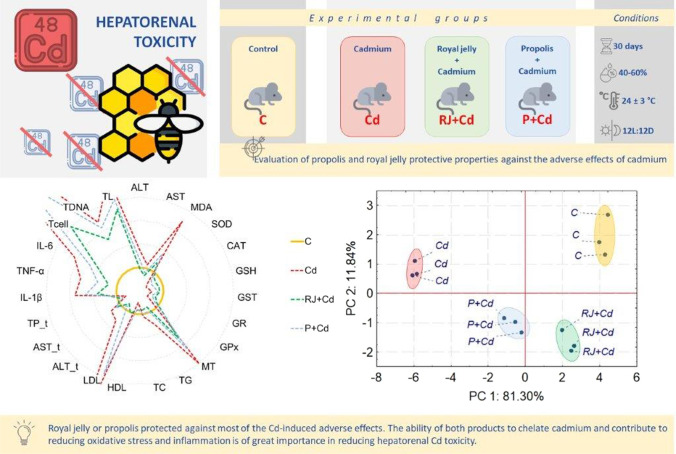

## Introduction

Heavy metal toxicity is an exponentially growing health problem. Of these heavy metals, cadmium (Cd) was reported to have disastrous effects on mammalian organs [1, 2]. Contaminated air, soil, drinking water, food, and cigarettes are the main sources of Cd exposure [3]. Occupational and environmental exposure to Cd leads to tissue degeneration, where the organ that suffers the highest soft tissue deposition of heavy metals, is the liver, followed by the kidneys [4, 5]. The mechanisms of the adverse effects of Cd exposure are relatively complex and have not yet been fully elucidated [6]. Nevertheless, two distinct pathways have been implicated in Cd toxicity, and the most well-known of which is the oxidative stress pathway [7]. Cd enhances reactive oxygen species (ROS) production, contributing to robust oxidative stress, which causes damage to proteins and other molecules, including DNA, disrupts their repair, and, finally, leads to cell dysfunction or death. Inflammation is also assumed as a significant contributor to Cd-induced tissue damage, where it promotes the upregulation of cell-secreted mediators and inflammatory markers and has pro-inflammatory properties. In addition to altering cellular function, Cd has been classified as a human carcinogenic agent [8].

Synthetic chelating agents, which are commonly used to bind and remove heavy metals from the body, have shown several adverse side effects. These side effects can incorporate gastrointestinal issues, allergic reactions, and disturbances in mineral balance. In contrast, natural antioxidant nutrients have become the future promise in heavy metal detoxification. A growing interest in bee products, including royal jelly (RJ) and propolis (P), helped their potential applications in the complementary and alternative medicine for several decades [9]. RJ is a milky secretion produced by worker bees’ hypopharine and submandibular glands to feed the developing queen bees. It is composed of proteins, monosaccharides, lipids, fatty acids, free amino acids (particularly essential amino acids including valine, methionine, leucine, threonine, phenylalanine, isoleucine, tryptophan, and lysine), minerals (including Na, Mg, Fe, Ca, Zn, Cu, K, and Mn), and vitamins (complexes A, B, E, and C) [10]. P is a complex mixture of resins collected by honey bees from various plant gums, then re-enriched with saliva and enzyme secretions and used to construct and protect beehives [11]. The chemical composition of P is complex and influenced by the botanical origin, the harvest, and the geographical origin [12]. Propolis has over 300 potentially active ingredients, including coumarins, phenolic aldehydes, steroids, amino acids, and polyphenols [13]. Due to its potential medicinal properties, P has been used for many different purposes, such as immune enhancement, antibacterial, anti-inflammatory, antitumor, and antioxidant entities [14, 15].

Taking the abovementioned information, we hypothesize that both substances or one of them can be effective in the mitigation of Cd adverse effects. Thus, this study aimed to investigate the protective effects of RJ and P to counter the health hazards caused by Cd accumulation in liver and kidney tissues in rats. To achieve the aim, many parameters characterizing the functions of the examined organs, the degree of Cd accumulation in tissues, and the level of oxidative stress and inflammation were examined. The histological slices of the liver and kidneys of rats exposed to Cd or RJ + Cd or P + Cd were also evaluated.

## Materials and Methods

### Chemicals

Cd as anhydrous CdCl_2_ was procured from Sigma-Aldrich (St. Louis, MO, USA). RJ powder was purchased from Staktich Inc. (Troy, MI, USA). P (Ref. POUPROP40) was obtained from Propolia, Apimab Laboratoires (Avenue du Lac, Clermont l'hérault, France). All chemicals were of high quality and analytical grade and used without further purification.

### Study Design

Thirty-two adult male albino rats were purchased from the Faculty of Medicine, Alexandria University, Egypt, and placed in the Department of Medical Physiology. They were housed under standard laboratory conditions (24 ± 3 °C, 40–60% humidity) and maintained on a 12-h light:12-h dark photoperiod cycle with free access to water and a standard chow diet (7% simple sugars, 3% fat, 50% polysaccharide, 15% protein (w/w), energy 3.5 kcal/g) supplied by local manufacturer (Tanta feed Company, Egypt). After acclimatization for 1 week, the animals were randomly divided into 4 groups of 8 rats each, receiving treatment and/or Cd dissolved in physiological saline (0.9% NaCl) by oral gavage for 30 days as follows: (1) The control group received physiological saline throughout the whole experiment duration; (2) the Cd group received a daily dose of 4.5 mg/kg body weight of CdCl_2_ [16]; (3) RJ+Cd group, rats were pre-treated with RJ (200 mg/kg/day) for 1 week [17, 18] before receiving CdCl_2_ simultaneously with the treatment after the 8th day; (4) P+Cd group, animals were pre-treated with P (50 mg/kg/day) for 1 week [19] before receiving CdCl_2_ simultaneously with the treatment after the 8th day until the end of the experiment. All experimental procedures were approved by the Research Ethics Committee, Faculty of Medicine, Alexandria University (protocol serial number: 0305766) and strictly followed all the procedures and techniques used to minimize any suffering during the experiments.

### Sample Preparation and Organs Collection

At the end of the experiment, rats were sacrificed under anesthesia by decapitation, and every effort was made to minimize suffering. Blood samples were taken from the retro-orbital venous plexus of the rat by inserting a capillary hematocrit tube under light ether anesthesia [20]. Blood was collected in clean, dry, non-heparinized Wassermann tubes for serum separation. The serum was separated by centrifugation at 700 × g for 15 min. Aliquots of serum were stored in Eppendorf tubes at −20 °C for measuring biochemical parameters. The liver and both kidneys were removed and then washed with ice-cold saline.

The left kidney was immediately homogenized in a cold phosphate buffer solution (PBS, 100 mM Na_2_HPO_4_/NaH_2_PO_4_, pH 7.4). The homogenates were centrifuged, and the resultant supernatants were transferred into Eppendorf tubes and preserved until use for biochemical evaluation. Meanwhile, the right kidney was isolated for Cd concentration determination and histopathological studies. The liver was dissected into two-halves, one-half was homogenized in cold PBS for biochemical analyses, and the other half was used for Cd concentration determination and histopathological investigation. A modification of the method of Lowry et al. was used for the determination of protein in the samples [21].

### Energy-Dispersive X-ray (EDX) Microanalysis to Determine Cd Content

The content of Cd was determined via EDX microanalysis using a scanning electron microscope (SEM). The liver and the kidneys were frozen at −70 °C and lyophilized at −35 °C for 24 h. The specimens were dried in a carbon dioxide critical point dryer, mounted in aluminum stubs, and coated with a thin layer of gold (≥20 nm) by a JFC−1100E−JEOL Ion sputter evaporator. They were analyzed and photographed at the Electron Microscope (EM) Unit, Faculty of Science, Alexandria University, Egypt, using the SEM at an accelerating voltage of 20 kV (JEOL JSM-5300, Tokyo, Japan). To obtain reliable analytical results, peaks were allocated automatically using SEM-EDX software for identification. The line intensities were measured for each element in the sample, as well as for the same elements using calibration standards of known composition. A stationary spot (×500) was detected randomly for 110 s.

### Biochemical Analyses

#### Determination of Liver Function Enzymes as Markers of Hepatic Injury

Both alanine transaminase (ALT) and aspartate aminotransferase (AST) were quantitatively assessed by the colorimetric method as recommended by the International Federation of Clinical Chemistry and Laboratory Medicine (IFCC; https://www.ifcc.org) [22] using Randox Laboratories diagnostics kits (London, UK) according to the manufacturer’s instructions.

#### Estimation of Kidney Function Markers

Serum urea and creatinine are the most widely accepted parameters to evaluate kidney dysfunction status. They were quantitatively assessed by the colorimetric method [23, 24] using Spectrum Diagnostic Kits, Egypt. Serum uric acid was determined spectrophotometrically using commercially available kits obtained from Spinreact diagnostic, Spain. Measurements of the above parameters were performed strictly according to the manufacturer’s instructions.

#### Determination of Serum Lipid Profile

Serum cholesterol (C) [25], serum triglycerides (TG), and high-density lipoprotein (HDL)-cholesterol (HDL-C) [26] were measured following manufacturing procedures (BioSystems S.A.). The low-density lipoprotein (LDL)-cholesterol (LDL-C) was calculated according to Friedewald’s equation [27].

#### Metallothionein (MT) Estimation in Liver and Kidneys

As one of the important features of CdCl_2_ toxicity is its ability to induce MT biosynthesis, the hepatic and renal MT concentration was quantitatively estimated by Rat Metallothionein ELISA kit (Cat. n^o^ CSB-E11315r) (CUSABIO Technology LLC., TX, USA) according to the manufacturer’s instructions.

#### Determination of Oxidative Stress Markers

Catalase (CAT) activity was determined as units per milligram of protein according to the method of Aebi [28]. Malondialdehyde (MDA) concentration was estimated as nanomoles per milligram of protein, according to Draper and Hadley [29]. Superoxide dismutase (SOD) was estimated as units per milligram of protein following the method of Marklund and Marklund [30]. Glutathione peroxidase (GPx) activity was determined as units per milligram of protein by the Flohe and Gunzler method [31]. Glutathione reductase (GR) activity was assayed as units per milligram of protein according to the method of Smith et al. [32]. Glutathione-S-transferase (GST) activity was determined as units per milligram of protein by the method modified by Carmagnol et al. [33].

#### Determination of Inflammatory Markers in Liver Tissues

Quantitative measurements of IL-1β (Cat. n^o^ MBS825017, MyBioSource Inc., CA, USA), IL-6 (Cat. n^o^ CSB-E04640r, CUSABIO Technology LLC., TX, USA), and TNF-α (Cat. n^o^ MBS824824, MyBioSource Inc., CA, USA) level were performed in liver homogenates using ELISA kits specified for rat according to the protocol provided with each kit.

### Estimation of DNA Damage by the Comet Assay

DNA damage was detected by the comet assay under alkaline conditions, following the procedure of Singh et al. [34]. The tissue was minced gently into tiny pieces in a chilled buffer solution consisting of 0.075 M NaCl and 0.024 M Na_2_EDTA using small dissecting scissors, then homogenized using a homogenizer. The cell suspension was centrifuged at 700 × g for 10 min at 4 °C, then re-suspended in a cold buffer, yielding a pellet. Cells were mixed with molten low melting point agarose, followed by the mixture spread over a frosted slide. The slides were placed in lysis solution for 60 min, followed by high pH electrophoresis (pH 13). Afterward, the slides were immersed in a neutralization buffer for 15 min. Samples were dried, stained with ethidium bromide, and viewed by an epifluorescence microscope (Leitz Orthoplan, Wetzlar, Germany) equipped with a 515–560-nm excitation filter and a 590-nm barrier filter for image analysis. The microscope was connected to a computerized image analysis system (Komet Assay V software, Perspective Instruments). Up to 100 randomly selected cells per slide were used to score comet. DNA damage was evaluated as tailed cells (TCell; %), tail DNA percentage (TDNA; %), and tail length (TL, μm) in each slide.

### Histopathological and Immunohistochemical Analyses

Liver and kidney tissue samples were fixed for 24 h at 25 °C in 10% neutral buffered formalin, dehydrated, embedded in paraffin, and sectioned (4–5 μm) using a rotary microtome, which were stained with hematoxylin and eosin (H&E), and examined using light microscopy.

Epitope retrieval was achieved by 3 min boiling in sodium citrate10 mM pH 6.0 at the microwave. After washing with PBS and blocking for 15 min with 5% bovine serum albumin in PBS with 0.1% Triton X-100, the presence of proliferating cell nuclear antigen (PCNA, cat. n^o^ ab218310, Abcam plc., Cambridge, UK) protein in liver samples was detected with a primary antibody against PCNA as a proliferative marker. Kidney samples were incubated at 4 °C overnight in a humidified chamber with a primary antibody against Wilms’ tumor suppressor gene 1 (WT1, cat. n^o^ ab224806, Abcam plc., Cambridge, UK), a protein necessary for normal kidney development. Primary antibodies were detected with the appropriate horse radish peroxidase (HRP)–conjugated secondary antibodies. HRP activity was detected with the chromogenic substrate ImmPACT DAB (SK-4105, Vector Labs, USA).

### Statistical Analysis

Before data analysis, the homogeneity of the variance and the normality of the data distribution were checked using the Levene test as well as the Kolmogorov-Smirnov and Lilliefors tests, respectively. The results of the tests confirmed ANOVA assumptions compliance. Therefore, the one-way analysis of variance, with the HSD Tukey (*p*< 0.05) as a post hoc test, was performed consecutively. Means ± SD were presented in charts. To visualize treatment effects on all parameters, radar charts were prepared, in which mean values were set as 100%, and all remaining means were recalculated accordingly. The results of the HSD Tukey test were marked with lowercase letters, where different letters denoted significant differences between experimental groups. Principal component analysis (PCA) was also performed to determine the relationship among measured parameters. The analysis covered variables and cases. All procedures were performed using Statistics 13.1. software.

## Results

### EDX Microanalysis to Determine Cd Content in the Hepatic and Renal Tissues of Rats

**Fig. 1:**
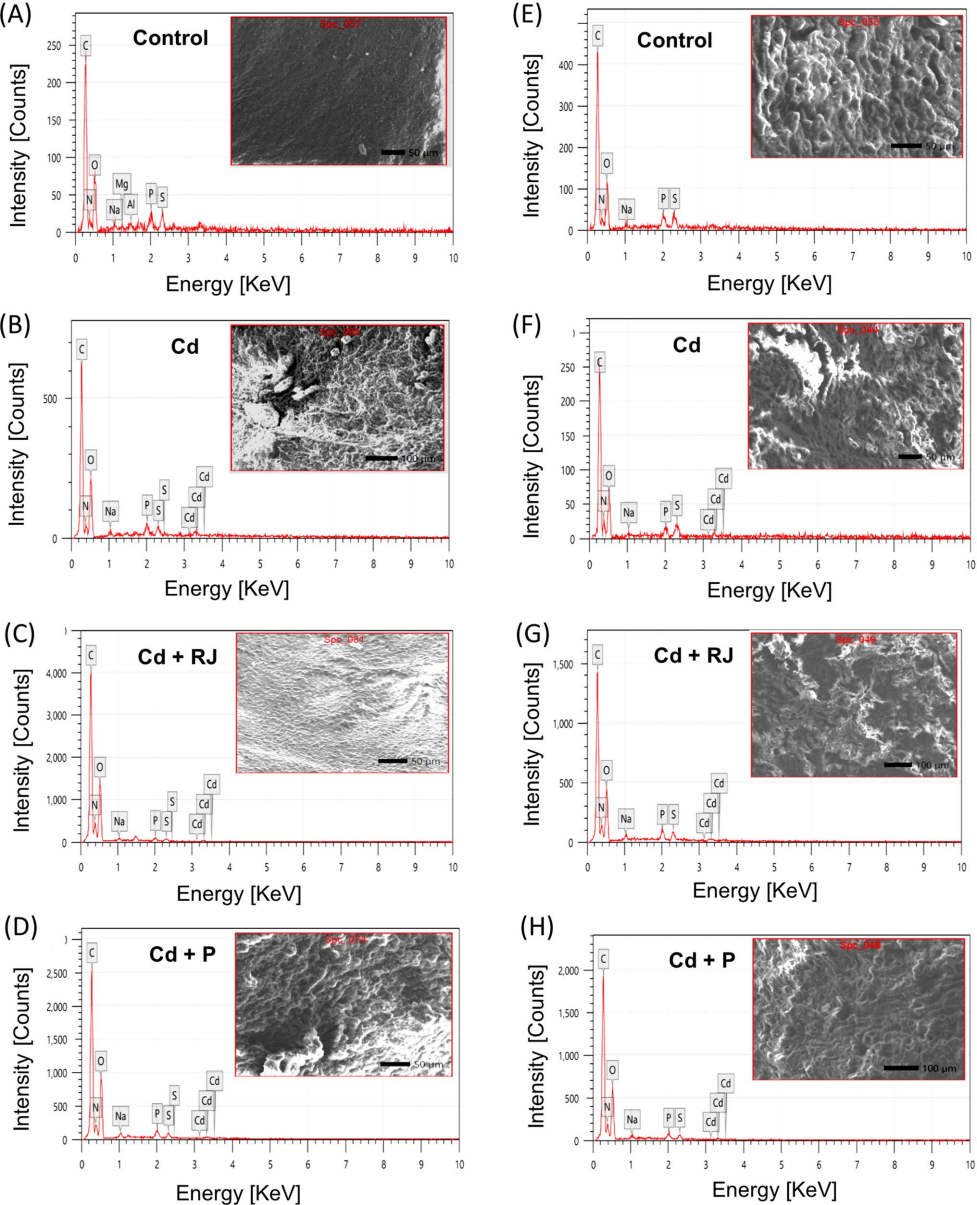
EDX spectra of selected area from the liver (**A**–**D**) and the kidney (**E**–**H**) in the different treated groups (SEM of the tissue inserted in the graphs), illustrating qualitative elemental composition (**A**–**H**). Abbreviations: C, carbon; N, nitrogen; O, oxygen; Na, sodium; P, phosphorus; S, sulfur; Cd, cadmium. Cd, rats receiving Cd as CdCl2 (4.5 mg/kg/day) during the experiment; RJ + Cd, rats pretreated with royal jelly (200 mg/kg/day) for 1 week and then simultaneously treated with royal jelly and cadmium; P + Cd, animals pretreated with propolis (50 mg/kg/day) for 1 week and then treated simultaneously with propolis and cadmium

Carbon (C), nitrogen (N), oxygen (O), sodium (Na), phosphorus (P), and sulfur (S) are the six basic elements that have been identified by EDX microanalysis in the hepatic (Fig. 1A–D) and renal tissues (Fig. 1E–H) of the control and the differently treated groups of rats. The percentage of the elements in both tissues is shown in Table 1. Cd was not detected in the tissues of rats from the control group, while in animals subjected to CdCl_2_ at a dose of 4.5 mg/kg, Cd was found in the tissues after 30 days. The mean percentage of Cd in the hepatic and renal tissues was 0.35 ± 0.11 and 0.27 ± 0.10, respectively. Interestingly, pretreatment with RJ at a dose of 200 mg/kg or with P at a dose of 50 mg/kg for 1 week and then treatment with RJ or P and Cd for up to 30 days causes a notable decrease in the mean percentage of Cd in the tissues compared to the CdCl_2_ treated groups, as shown in Table 1. No significant difference was found between RJ and P.

**Table 1:** Illustrate quantitative elemental composition as well as Cd content in the hepatic and renal tissues of the different treated groups using EDX microanalysis

Liver (mass%)					Kidney (mass%)			
Experimental groups								
Element	Control	Cd	RJ + Cd	P + Cd	Control	Cd	RJ + Cd	P + Cd
C	53.60±0.68	50.18±0.45	45.48±0.19	47.11±0.22	51.18±0.50	48.72±0.46	48.00±0.27	46.05±0.20
N	11.95±1.20	13.96±0.87	17.82±0.41	14.63±0.47	14.88±0.98	17.71±0.99	17.72±0.57	18.75±0.44
O	30.48±1.25	33.24±0.95	35.31±0.44	35.78±0.49	31.05±1.01	30.78±0.98	31.47±0.55	33.69±0.46
Na	0.64±0.13	0.49±0.08	0.46±0.04	0.76±0.05	0.67±0.10	0.53±0.09	0.68±0.06	0.38±0.04
P	1.56±0.14	1.03±0.09	0.45±0.03	0.96±0.04	1.23±0.10	1.00±0.09	1.09±0.05	0.56±0.03
S	1.49±0.13	0.79±0.07	0.31±0.06	0.53±0.03	0.93±0.09	0.99±0.08	0.74±0.04	0.51±0.03
Cd	nd	0.35±0.11	0.05±0.03	0.07±0.04	nd	0.27±0.10	0.02±0.01	0.07±0.03

Abbreviations: *C*, carbon; *N*, nitrogen; *O*, oxygen; *Na*, sodium; *P*, phosphorus; *S*, sulfur; *Cd*, cadmium; *nd*, not detected. *Cd*, rats receiving Cd as CdCl2 (4.5 mg/kg/day) during the experiment; *RJ + Cd*, rats pretreated with royal jelly (200 mg/kg/day) for 1 week and then simultaneously treated with royal jelly and cadmium; *P + Cd*, animals pretreated with propolis (50 mg/kg/day) for 1 week and then treated simultaneously with propolis and cadmium

### Analyses of Liver Biochemical Parameters

#### Impacts of RJ and P on Serum Liver Enzymes in Rats Supplemented with Cd

**Fig. 2:**
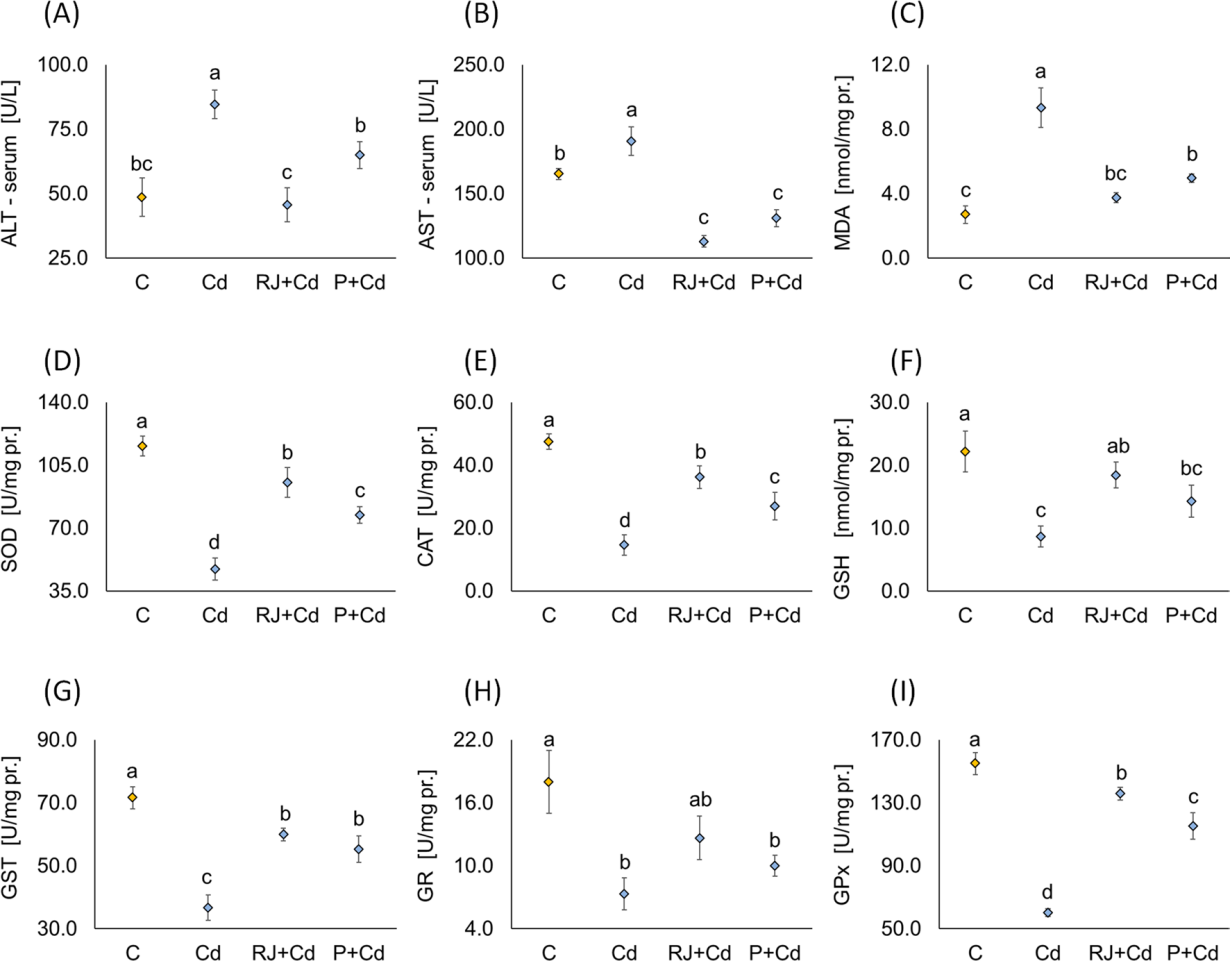
Determination of liver function enzymatic activities (**A**, **B**), oxidative stress intensity (**C**), and antioxidant enzymes activities (**D**–**I**) in control male rats versus animals receiving different treatment protocols for 30 days. Data are represented as mean ± SD. The same letter (a, b, c, d) denotes no significant difference among experimental groups tested within each parameter separately (*p*<0.05; *n*=5; LSD, ANOVA test). Abbreviations: ALT, alanine transaminase; AST, aspartate aminotransferase; MDA, malondialdehyde; SOD, superoxide dismutase; CAT, catalase; GSH, glutathione; GST, glutathione S-transferase; GR, glutathione reductase; GPx, glutathione peroxidase; other explanations, see Fig. 1

Liver function tests revealed a significant increase in serum ALT and AST in rats receiving Cd as CdCl_2_ (4.5 mg/kg/day) for 30 days compared to the controls (Fig. 2A, B). Increased ALT and AST activities can be attributed to the leakage of these enzymes from the liver cytosol into the bloodstream. Such an increase was significantly lowered by RJ and P pretreatments. RJ reduced serum ALT more significantly than P pretreatment.

#### Effects of RJ and P on Cd-Induced Oxidative Stress and Antioxidant Enzymes

There is a wide range of xenobiotics whose toxicity is associated with the intensification of oxidative stress. Cd administered as CdCl_2_ to male rats mediated high levels of MDA (Fig. 2C) and lowered the GSH concentration as well as the antioxidant enzymes activities (Fig. 2D–I). RJ and P brought MDA level, GSH concentration, and other antioxidant enzyme activities almost to standard/reference values. RJ showed a more potent antioxidant effect manifested by a significant increase in liver SOD, CAT, and GPx than P.

#### Effects of RJ and P on the Physiopathology of Liver Tissue Parameters, Serum Lipid Profile, and MT Level in Rats Supplemented with Cd

**Fig. 3:**
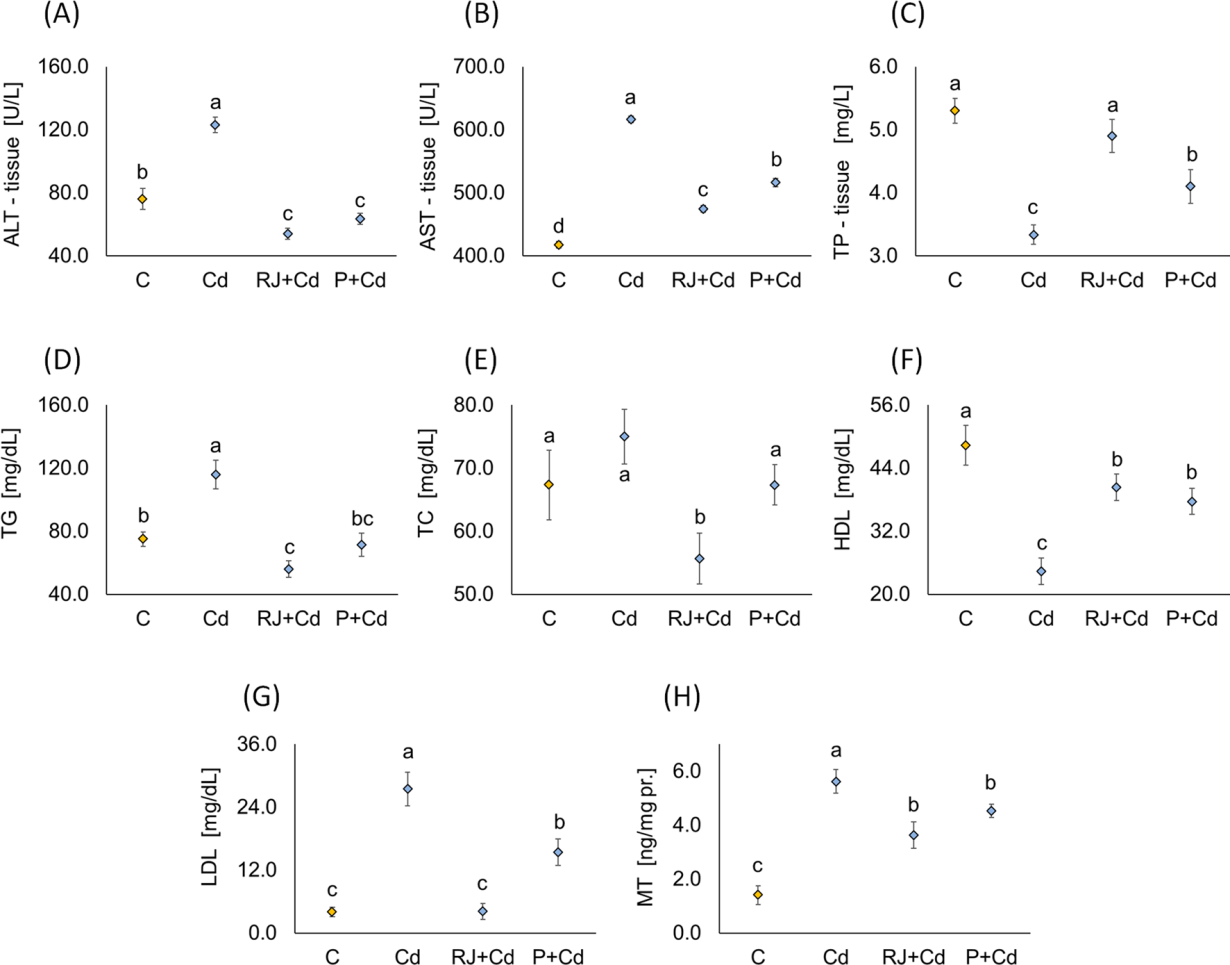
Mean ± SD of alanine transaminase (**A**), aspartate aminotransferase (**B**), and total protein (**C**) in the liver tissue, triglycerides (**D**), total cholesterol (**E**), high-density lipoprotein (**F**), low-density lipoprotein (**G**) in the serum, and metallothionein (**H**) of the different experimental male rats treated for 30 days. The same letter (a, b, c, d) denotes no significant difference among experimental groups (*p*<0.05; *n*=5; LSD, ANOVA test). Abbreviations: ALT_tissue, alanine transaminase measured in liver tissue; AST_tissue, aspartate aminotransferase measured in liver tissue; TP_tissue, total proteins; TG, triglycerides; TC, total cholesterol; HDL, high-density lipoprotein (HDL) cholesterol; LDL, low-density lipoprotein (LDL) cholesterol; MT, metallothionein; other explanations, see Fig. 1

Compared to the negative influence of Cd on liver tissue parameters, our results showed a significant decrease of ALT and AST in RJ+Cd and P+Cd groups (Fig. 3A, B). This decrease is more drastic for ALT than AST compared to Cd-treated rats and the corresponding controls, even though there was no significant difference between RJ- and P-treated groups regarding tissue ALT.

Moreover, Cd significantly diminished the total protein level compared to control rats (Fig. 3C). Conversely, these levels showed a significant rise following RJ and P pretreatments compared to Cd-treated animals. However, RJ showed a higher protective effect than P, which increased the total protein levels significantly, reaching levels similar to that of control animals.

We assessed the lipid profile in the different experimental groups to complete the global picture concerning the adverse effects of Cd administration upon liver dysfunction and how it could be alleviated via bee natural products treatment. The lipid profile showed considerable deterioration in Cd-treated rats, where these animals showed a significant increase in total TG and LDL-cholesterol and a significant decline in HDL-cholesterol compared to control rats (Fig. 3D–G). However, RJ and P administration reversed that deterioration, where they caused a significant drop in total TG and LDL-cholesterol and a significant rise in HDL-cholesterol compared to Cd-treated rats. RJ and P effects on total TG and HDL were almost the same, while RJ showed a more significant decline in LDL-cholesterol than P. P brought serum TG back to normal, where there was no significant difference between P-treated rats and controls. On the other hand, RJ could normalize serum LDL-cholesterol in RJ-treated rats as there was no significant difference between control and RJ-treated rats. Regarding total cholesterol, there was no significant difference between control, Cd, and P-treated rats. However, RJ-treated males manifested a significant decrease in the total cholesterol level (Fig. 3E).

Hepatic MT expression is a valuable marker for pathogenicity in the liver. Its level was significantly higher in Cd-treated rats compared to controls (Fig. 3H). Conversely, RJ and P treatment caused a significant decrease in hepatic MT levels compared to Cd rats. There was no significant difference between RJ+Cd and P+Cd groups regarding their effect on hepatic MT.

#### Effects of RJ and P on Cd-Mediated Inflammatory Markers

**Fig. 4:**
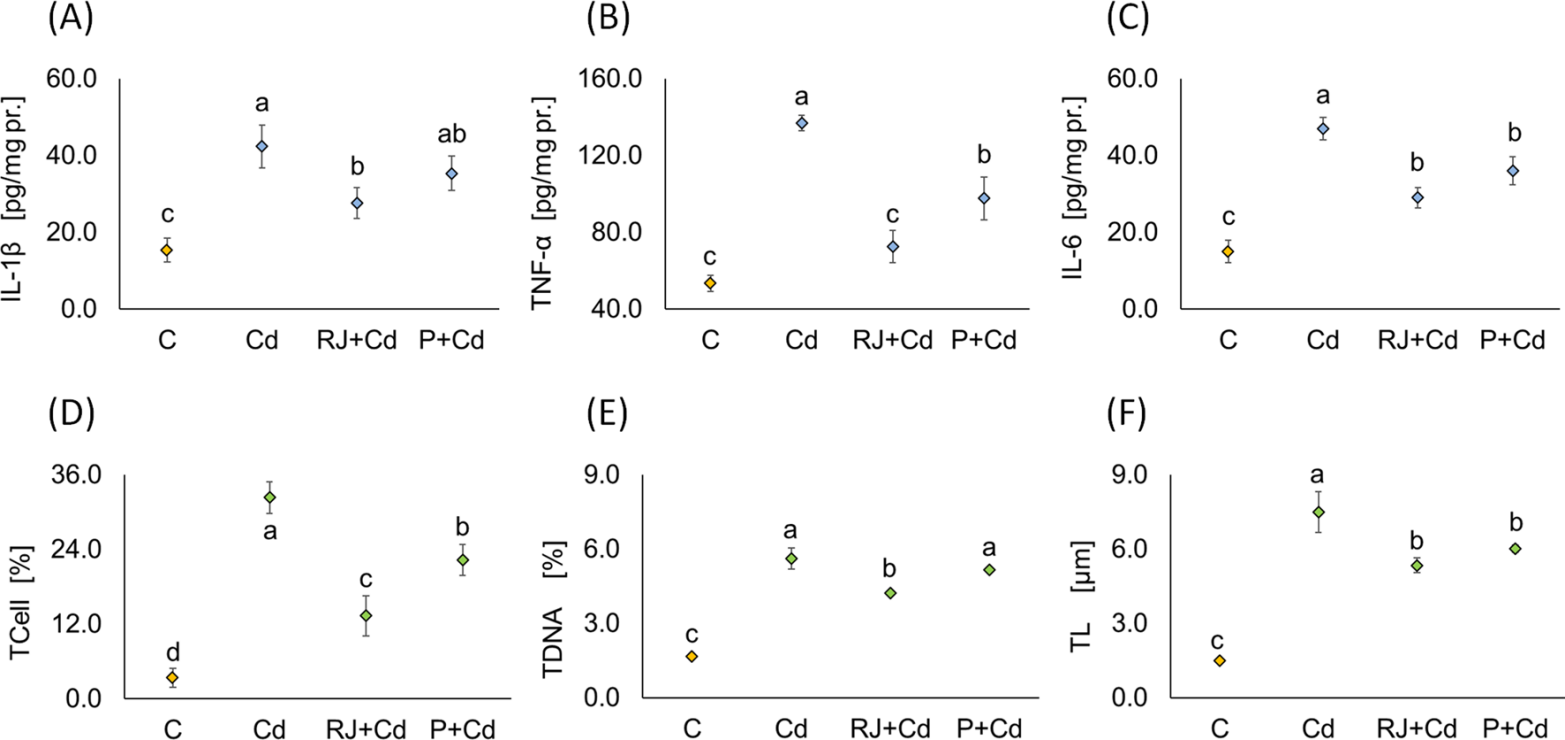
Mean ± SD of interleukin-1β (**A**), tumor necrosis factor α (**B**), and interleukin-6 (**C**) in liver of control male rats versus animals receiving different treatment protocols for 30 days. Assessment of DNA damage using comet assay (**D**–**F**), data are also represented as mean ± SD. The same letter denotes no significant difference among experimental groups tested within each parameter separately (*p*<0.05; *n*=5; LSD, ANOVA test). Abbreviations: IL-1β, interleukin-1β; TNFα, tumor necrosis factor α; IL-6, interleukin-6; TCell, tailed cell; TDNA, tail DNA; TL, tail length; other explanations, see Fig. 1

Administration of Cd to rats has a significant influence on the concentrations of IL-1β (42.3 ± 5.5), TNF-α (137.0±4.0), and IL-6 (47.0±3.0) in the liver when compared to controls (15.3±3.1, 53.3±4.2, and 15.0±3.0, respectively) as shown in Fig. 4A–C. Compared to Cd-treated males, RJ-treated animals showed a significant reduction in these inflammatory markers (IL-1β (27.7±4.0); TNF-α (72.7±8.3); IL-6 (29.0±2.6)), while P pretreatment resulted in a significant decrease in IL-6 (36.0±3.6) and TNF-α (97.7±11.2), but not in IL-1β (35.3±4.5). RJ and P effects displayed almost the same concerning IL1β and IL6. However, RJ showed a stronger inhibitory effect on TNF-α than P. Compared to controls, the RJ pretreatment restored liver TNF-α to standard levels with no significant difference (Fig. 4B).

#### Effects of RJ and P on Cd-Induced DNA Damage

DNA damage was manifested in Cd-treated rats by a significant increase in tailed cells (Fig. 4D), tail DNA percentage (Fig. 4E), and tail length (Fig. 4F). Both RJ and P pretreatments ameliorate DNA damage. However, RJ pretreatment revealed better positive effects, especially regarding the tailed cells and tail DNA percentage parameters.

#### Relationship Among All Measured Parameters in the Liver of Rats

**Fig. 5:**
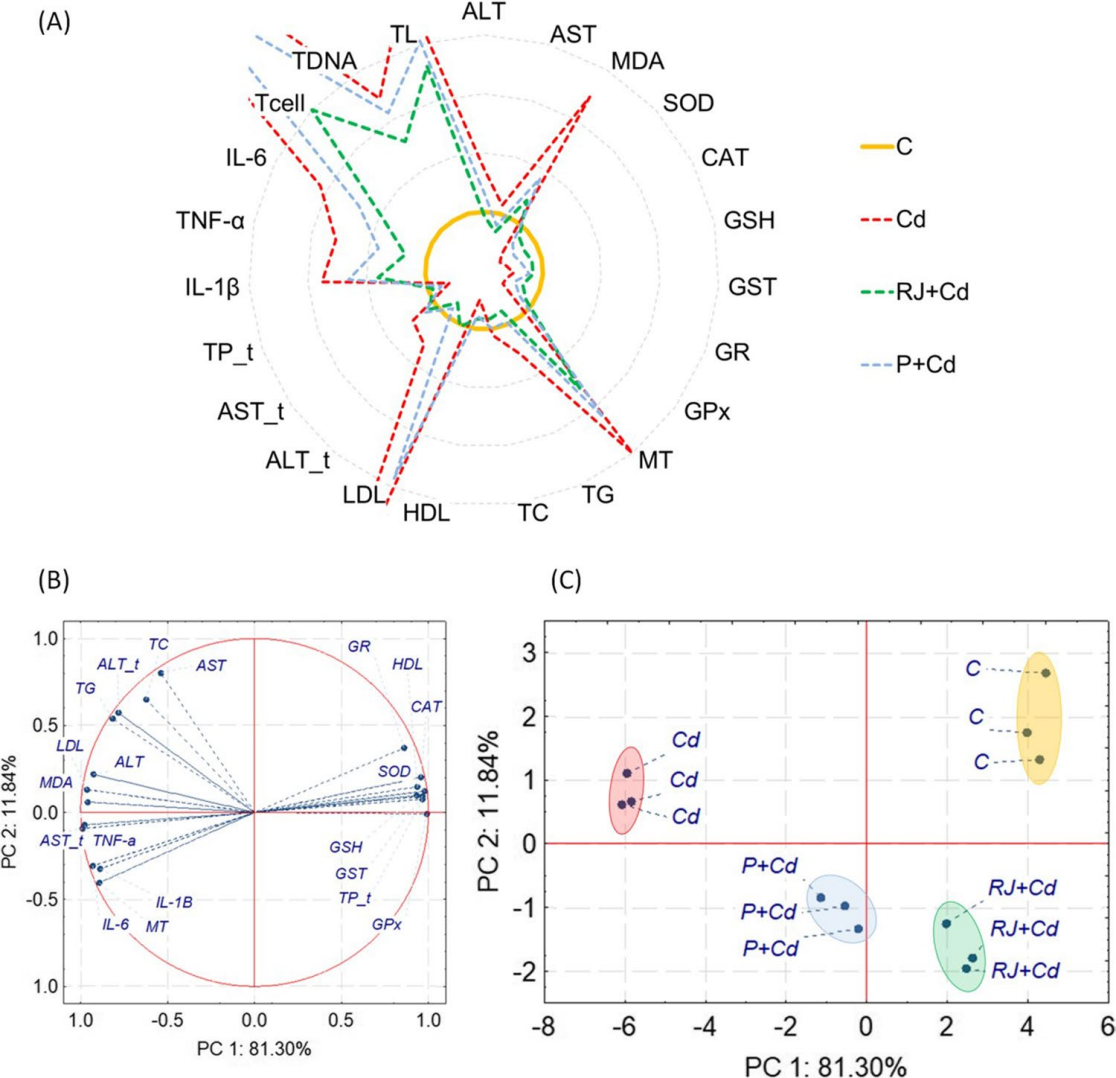
Relationship among all measured parameters in the liver of rats. **A** Radar chart, where means for the control group were set as 100%, and the remaining means were recalculated accordingly; **B** principal component analysis on all measured markers; and **C** 2D principal component analysis plot of control and experimental groups cases. Abbreviations: ALT, alanine transaminase measured in the serum; AST, aspartate aminotransferase measured in the serum; TG, triglycerides; TC, total cholesterol; HDL, high-density lipoprotein (HDL) cholesterol; LDL, low-density lipoprotein (LDL) cholesterol; ALT_t, alanine transaminase measured in liver tissue; AST_t, aspartate aminotransferase measured in liver tissue; TP_t, total proteins; IL1-β, interleukin-1β; TNF-α, tumor necrosis factor α; IL-6, interleukin-1β; Tcell, “tailed cells” — cells with damaged DNA measured by the comet assay; TDNA, the amount of DNA in the comet tail; TL, the length of the tail comet; MDA, malondialdehyde; SOD, superoxide dismutase; CAT, catalase; GSH, reduced glutathione; GST, glutathione S-transferase; GR, glutathione reductase; GPx, glutathione peroxidase; MT, metallothionein; PC1, first principal component; PC2, second principal component; other explanations, see Fig. 1

All markers measured in the liver were significantly affected by Cd, as shown in the radar chart (Fig. 5A). Similarly, oxidative stress parameters decreased significantly after Cd treatment, and MT and MDA increased dramatically compared to the control animals. Additionally, the LDL level was elevated by six times, and the number of cells with damaged DNA increased by almost ten times, forming comets with bigger (more than three times) and longer (5 times) tails than in the control group. Pretreatment with RJ gave more beneficial effects than P (Fig. 5A — compare yellow control line with RJ+Cd green line). PCA explained as much as 93.14% of liver markers variance. Oxidative stress markers, liver function parameters, LDL, and HDL mainly created PC1. The PC2 was associated with AST, TC, ALT_t, TG, inflammatory markers, and MT (Fig. 5B). 2D PCA plot revealed four separated clusters, where the cluster for the cadmium group was at the longest distance from the control cluster. The PCA analysis confirmed the beneficial effect of propolis or royal jelly on the liver. Similarly, like for kidneys, royal jelly perceived better outcomes than propolis. However, the control group was still visibly separated from the RJ+Cd group (Fig. 5C).

### Analyses of Kidney Biochemical Parameters

#### Impacts of RJ and P on Renal Function Tests in Rats Treated with Cd

**Fig. 6:**
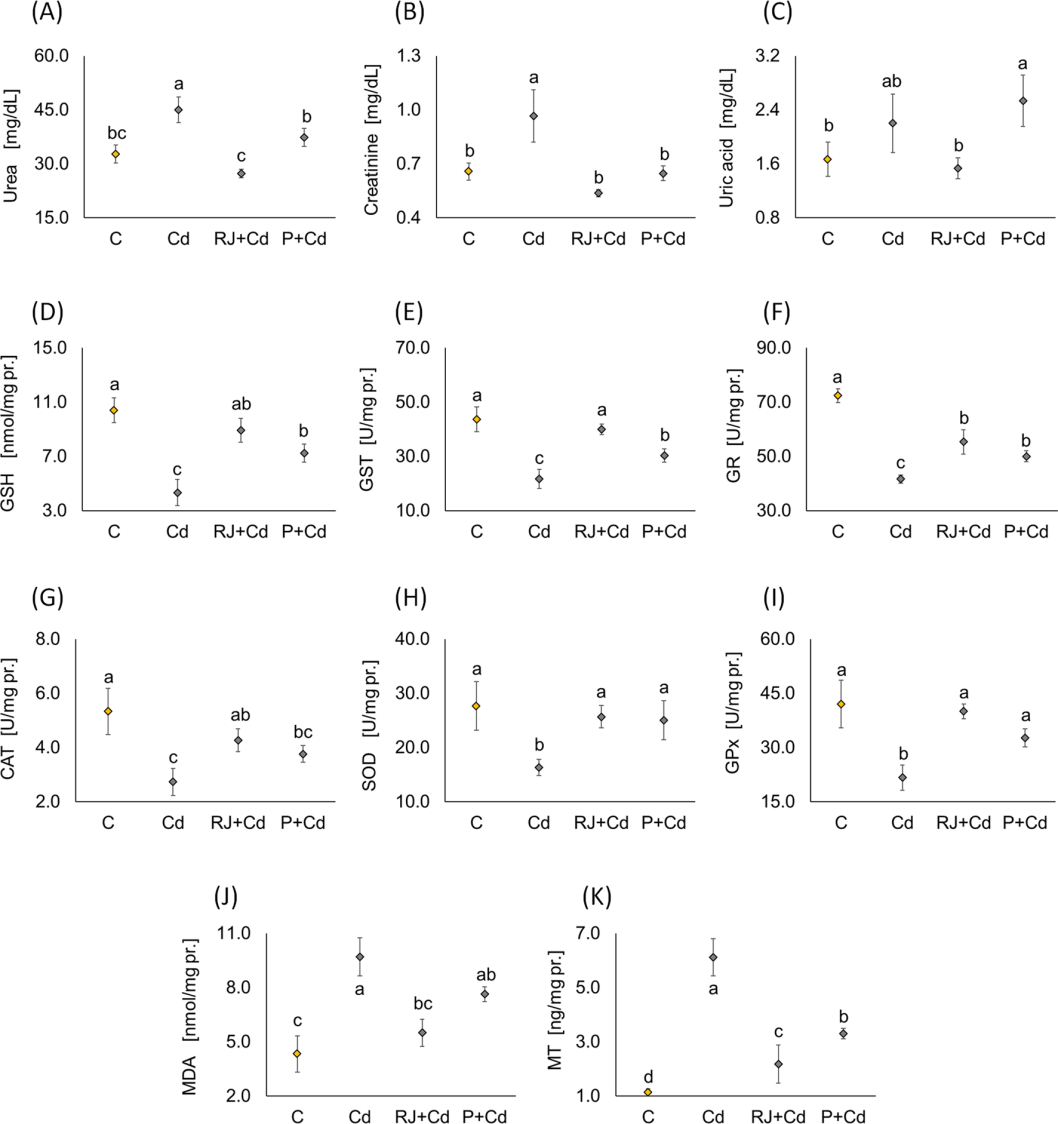
Determination of biochemical renal parameters including urea (**A**), creatinine (**B**), uric acid (**C**), antioxidant parameters (**D**–**I**), oxidative stress (**J**), and metallothiomein (**K**) in control male rats versus animals receiving different treatment protocols for 30 days. Data are represented as mean ± SD. The same letter (a, b, c, d) denotes no significant difference among experimental groups tested within each parameter separately (*p*<0.05; *n*=5; LSD, ANOVA test). Abbreviations: GSH, glutathione; GST, glutathione S-transferase; GR, glutathione reductase; CAT, catalase; SOD, superoxide dismutase; GPx, glutathione peroxidase; MDA, malondialdehyde; MT, metallothionein; other explanations, see Fig. 1

Compared to the control group, Cd-treated rats showed deterioration of renal function parameters illustrated by a significant elevation in serum urea and creatinine (Fig. 6A, B). On the other side, rats receiving RJ or P showed an improvement in renal function with a significant decrease in serum urea and creatinine compared to Cd groups, reaching almost reference levels where there was no significant difference between the treated and control groups. Regarding uric acid, there was no significant difference between Cd and control groups (Fig. 6C). Interestingly, there was a significant increase in serum uric acid in the P group compared to the control and RJ groups. Additionally, there was no significant difference between P and Cd rats.

#### Effects of RJ and P on Cd-Induced Oxidative Stress Parameters

Regarding the oxidative profile in the kidney, Cd-treated rats showed a lower level of GSH (Fig. 6D) and decreased activities of kidney oxidative stress enzymes, including GST, GR, CAT, SOD, and GPx (Fig. 6E–I) than in control rats. RJ cotreatment with Cd caused a significant increase in oxidative stress enzymes compared to Cd-treated rats, almost reaching the level of control animals. P also caused a significant increase in these enzymes except renal CAT. Additionally, RJ pretreatment nearly normalized renal GSH, GST, CAT, SOD, and GPx. On the other side, P could only normalize renal SOD and GPx.

#### Effects of RJ and P on Cd-Mediated Antioxidants

MDA, the final product of polyunsaturated fatty acids peroxidation, was significantly higher in the Cd group (Fig. 6J). Renal MT also increased significantly in the Cd group compared to control rats. RJ and P cotreatment resulted in a significant decrease in both parameter levels compared to Cd-treated rats. Again, RJ showed a better protective effect than P. Nevertheless, both treatments did not bring renal MT levels back to the control level.

#### Relationship Among All Measured Parameters in the Kidney of Rats

**Fig. 7:**
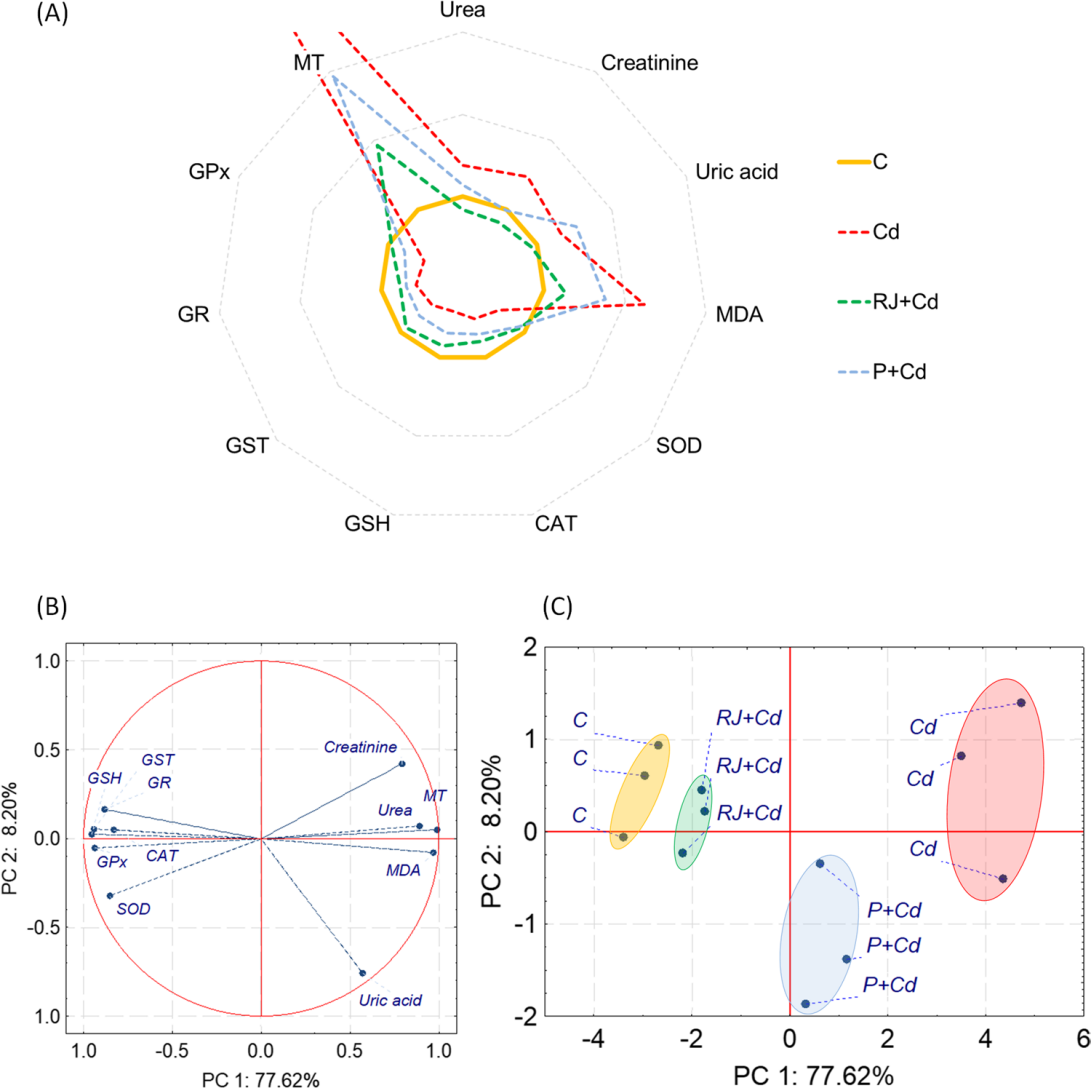
Relationship among all measured parameters in the kidney of rats. **A** Radar chart, where means for the control group were set as 100% and the remaining means were recalculated accordingly; **B** principal component analysis on all measured markers; and **C** 2D principal component analysis plot of control and experimental groups cases. Abbreviations: see Figs. 1 and 6

Like in the liver, cadmium treatment influenced all measured kidney parameters (Fig. 7A). Oxidative stress markers were significantly lowered compared to the control animals. In contrast, MT and MDA were increased by more than 5 and 2 times, respectively. Also, urea, uric acid, and creatinine were elevated in the cadmium group compared to the control one. The treatment with RJ revealed better effects than P, bringing most measured parameters closer to the control ones (Fig. 7A — compare yellow control line with RJ + Cd green line). PCA calculated for kidney parameters revealed the importance of the first component, PC1, which explained most of the data variance (77.62%). PC1 was negatively associated with urea, MT, and MDA and positively related to oxidative stress markers (Fig. 7B). PC2 explained only 8.20% of data variability and was influenced mainly by uric acid and creatinine. All types of treatment affected the measured parameters differently. Still, the cadmium treatment had the most potent effect, creating a detached cluster distinctly separated from the control group and located on the opposite side of the 2D PCA plot (Fig. 7C). Cotreatment of animals with P or RJ and cadmium alleviated the adverse effects to some extent. The PCA analysis confirmed that the positive impact was more remarkable for RJ than P. Still, clusters for both cotreated groups remained distinct from the control one (Fig. 7C).

#### Effects of RJ and P on Hepatic and Renal Histopathology in Rats Supplemented with Cd

**Fig. 8:**
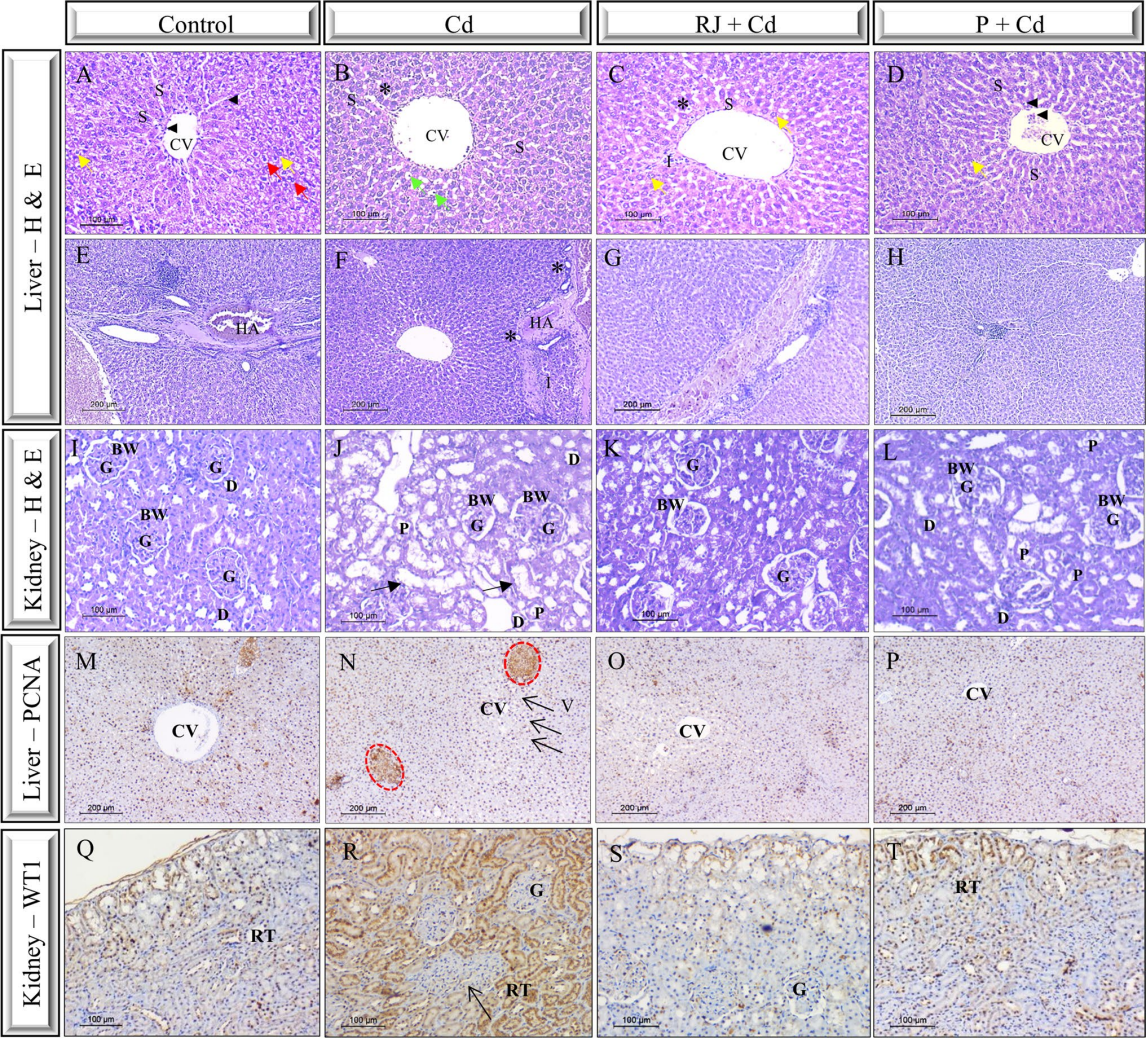
Representative photomicrographs of liver (**A**–**H**; **M**–**P**) and kidney (**I**–**L**; **Q**–**T**) sections of different experimental groups. H&E-stained liver samples showing (i) normal hepatic architecture in controls (**A**, **E**) within which arrowheads indicate flattened endothelial cells, red arrows demarcate binucleated hepatocytes, and yellow arrows distinguish rounded vesicles; (ii) vacuolation of hepatocytes with sinusoidal dilation and disorganization of hepatic cords in Cd group (**B**, **F**) within which green arrows indicate necrosis and the asterisks show cytoplasmic vacuolation; (iii) RJ+Cd group (**C**, **G**) with rounded vesicles (yellow arrows) and cytoplasmic vacuolation (asterisk); and (iv) P+Cd group (**D**, **H**) showing flattened endothelial cells (arrowheads) and rounded vesicles (yellow arrow). H&E-stained kidney samples illustrate the basic renal structures (**I**–**L**), note that the arrows indicate the degeneration of the epithelial lining of the renal tubules in Cd-treated rats (**J**). Immunohistochemical staining with PCNA of liver sections (**M**–**P**) depicting focally positive cells (brown) arranged in a trabecular pattern in Cd group (**N**), encircled by red broken line. Note the presence of numerous vacuoles (arrows). Immunohistochemical staining with WT1 of kidney sections (**Q**–**T**) illustrating the elevated expression in Cd group (**R**). Note the presence also of fused glomeruli (arrow). Abbreviations: CV, central vein; HA, hepatic artery; I, inflammation infiltration; S, hepatic sinusoid; BW, Bowman’s capsule; D, distal tubules; G, glomerulus; P, proximal tubules; RT, renal tubule; PCNA, proliferating cell nuclear antigen; WT-1, Wilms’ tumor suppressor gene 1

Examination of liver samples showed normal histoarchitecture in control rats (Fig. 8A, E). However, the hepatocytes lost their characteristic regular arrangement of hepatic strands in the Cd group (Fig. 8B, F). Most hepatocytes exhibited vacuolation, and their nuclei were partially pyknotic. Degeneration of hepatocytes and sinusoidal dilution were also observed in the Cd-treated rats. Interestingly, pretreatment with RJ for 1 week before Cd administration reversed most of the previously observed histopathological alteration inflected by Cd, except for some vacuoles (Fig. 8C, G). Also, P+Cd-treated rats displayed characteristic hepatocyte organization; the nuclei were vesicular and showed normal appearance like controls (Fig. 8D, H).

Regarding the kidney structure, the renal cortex and medulla were normal in appearance in the control group (Fig. 8I). In contrast, in Cd-induced rats, renal corpuscles showed extensive congestion filling the glomerular capillary loops, some glomeruli were atrophied, and Bowman’s space was widened. The histological deterioration was also characterized by degeneration, occurring primarily in proximal convoluted tubules and progressive deformation of capillary endothelial cells. In addition, inflammatory cell infiltrations were clearly apparent after Cd induction (Fig. 8J). More importantly, these histological abnormalities were visibly reduced in RJ+Cd and P+Cd groups (Fig. 8K, L).

In an attempt to confirm the pathology mediated by cadmium administration, we analyzed PCNA expression in the liver sections of the different experimental groups. Cd exposure revealed focally positive staining showing the cells arranged in a trabecular pattern (Fig. 8N). Such an aberrant manifestation was not observed in RJ+Cd (Fig. 8O) and P+Cd (Fig. 8P) groups. Furthermore, PCNA positively stained cells in RJ+Cd and P+Cd groups were lessened compared to the Cd group (Fig. 8N) and were more likely similar to control group (Fig. 8M). While WT1 expression was highly detected in the renal tubules of rats treated with Cd (Fig. 8R) as compared to controls (Fig. 8Q) or RJ+Cd (Fig. 8S) and P+Cd (Fig. 8T) animals.

## Discussion

Cd is considered one of the naturally toxic elements that can have severe effects on humans and animals. Continuous exposure to Cd causes its accumulation mainly in the metabolically dynamic tissues, such as the liver and kidney. In the present study, an attempt was made to assess the impact of Cd toxicity on the liver and kidney, followed by investigating the possible role of RJ and P co-administration to ameliorate the toxic effect. Histological and biochemical assessment was used for the evaluation of hepatic and renal damage resulting from Cd toxicity.

Repeated exposure to Cd, a non-biodegradable metal, induces the synthesis of MT in the liver [35]. MT is a small protein; one-third of its amino acid residues are cysteines. These cysteine residues bind and store metal ions and play a substantial role in Cd immobilization [36]. Cd combines with MT mainly in the liver. Subsequently, a small proportion of liver Cd/MT enters plasma from where it is filtered through the glomerular membrane and taken up in kidney tubules where cellular damage may ensue [35]. In this study, elevated levels of MT were observed in the Cd group, whereas their levels significantly decreased in RJ and P cotreated rats. This suggests a potential protective activity of RJ and P.

In the current study, liver injury was evident in the Cd group, manifested by decreased total protein levels, increased liver enzymes, AST, and ALT, and disturbed lipid profile. These results are in agreement with earlier studies [37, 38]. Recent reports have suggested that lysosome instability caused by Cd toxicity resulted in the leakage of hepatic enzymes, including ALT and AST, into the bloodstream [39]. Histological examination of liver tissue showed liver injury in the Cd-treated group, where hepatocytes lost their characteristic regular arrangement. Most hepatocytes exhibited vacuolation, and their nuclei were partially pyknotic. In contrast, RJ and P cotreatment showed hepatoprotective effects, proven by restoration of liver structure and function in these groups. The hepatoprotective effects of RJ observed in this study are in complete agreement with Almeer et al. [40], supporting the notion that RJ restored liver architecture and function following cadmium toxicity.

Concerning kidney functions, the increased levels of biomarkers in our study reflected the deterioration of the kidney functions following Cd exposure. Histopathological assessment of kidney tissue showed degeneration, occurring primarily in proximal convoluted tubules epithelia and progressive deformation of capillary endothelial cells. In addition, inflammatory cell infiltrations were clearly apparent after Cd induction. These histological abnormalities were found to be reduced in RJ+Cd and P+Cd groups. The cotreatments with RJ and P led to almost complete restoration of the Cd-induced renal damage and restored the standard values of the kidney function markers. These results were consistent with Almeer et al. [37], in which RJ restored kidney structure and function following cadmium toxicity.

Cd-induced hepatorenal dysfunction in this study was most likely due to an imbalance of oxidant/antioxidant capacity in the tissues. The present study showed a significant increase in MDA levels and a decrease in the activity of antioxidant enzymes, such as SOD, GPx, CAT, GR, GST, and GSH, in both hepatic and renal tissues of Cd-induced rats. The endogenous antioxidants are important scavengers of free radicals, which eventually lead to cellular dysfunction and death [41]. Our findings agreed with previous reports demonstrating that cadmium induces kidney toxicity by enhancing ROS formation and GSH consumption and inhibiting an antioxidant-mediated defense system [42].

The present study suggested that the administration of RJ and P with cadmium ameliorates the alteration in enzymatic activity of antioxidant molecules and prevents the generation of free radicals, further reducing Cd-induced oxidative damage. Furthermore, previous studies reported that the antioxidant activity of RJ is not only due to the hydroxyl radical-scavenging activity but also due to the indirect effect of RJ based on the suppression of enzymes that enhance the peroxidation of endogenous lipids as well as cytochrome P450 expression, which is one of the intracellular sources of oxygen radicals [40]. Furthermore, RJ contains free amino acids such as methionine, proline, cysteine, and cystine, which might be responsible for this effect by promoting biosynthesis of glutathione and scavenging free radicals, in addition to the ability of RJ to enhance the activity of antioxidant enzymes [43]. Additionally, the nephroprotective effect of P was owned to the improvement of renal oxidation and decrease of serum creatinine urea levels which are in consistence with our results [44]. Promsan et al. [45] studied one of the main constituents of P flavonoids, pinocembrin (5,7-dihydroxyflavone), which enhanced renal function and diminished apoptotic and oxidative stress markers.

In the current study, Cd administration induces an elevation of IL-1β, IL-6, and TNF-α in rats. TNF-α is a cell signaling cytokine involved in systemic inflammation by firing up the acute phase reaction. TNF-α can activate three diverse pathways: mitogen-activated protein kinase (MAPK), NF-κB, and cell death signaling pathways. The current results agree with the previously reported results [46]. In this study, RJ and P diminished the inflammatory markers’ elevation, mainly due to their phenolic mixes and other minor constituents [47, 48]. The anti-inflammatory activity of RJ was reported, and one of the major lipid constituents in RJ is 10-hydroxy-2-decenoic acid, which was said to exert anti-inflammatory consequences by inhibiting NF-κB. Caffeic acid phenethyl ester (CAPE), the main constituent in P, may be responsible for propolis’ anti-inflammatory effects by lowering the inflammatory cytokines in cells [47, 48]. It is worth mentioning that inflammation may complicate the pathologic processes of oxidative stress, adding deleterious effects to hepatorenal injury. Therefore, RJ and P would play a vital protective role against hepatorenal damage in case of Cd toxicity.

DNA damage assessed by the comet assay is considered an indication of molecular changes, which may lead to pathological alterations [49]. In this study, Cd-induced DNA damage in the liver was evidenced by a significant increase in tail DNA percentage, tailed cells, and tail length. In comparison, both RJ and P treatments succeeded in reducing Cd-induced damage, an effect that can likely be associated with reducing oxidative stress by the tested bee products. Thus, the properties of RJ and P may cause some of the Cd to be retained, preventing further adverse effects usually induced by toxic metals.

In the current study, the observed Cd-induced hepatorenal histopathological abnormalities are consistent with previous results depicting similar alterations in rats treated with Cd administered as CdCl_2_ where the dose was 5 mg/kg animal for 28 days in the case of liver and it was 6 mg/kg animal for 8 weeks in the case of kidney [50, 51]. Here we also observed that the proliferation of hepatocytes, as manifested by PCNA expression, exhibited focally positive cells arranged in a trabecular pattern in Cd-treated rats. Similarly, WT1 expression was highly detected in the renal tubules. The elevated expression of WT1 mediated by cadmium administration was neutralized by the pretreatment with RJ and P. Most importantly, our study has shown that both RJ and P pretreatments possess potent hepatorenal protective and antioxidative properties. Conforming to previous investigations, we revealed that RJ and P could successfully alleviate the adverse effects of Cd on liver and kidney histoarchitecture [37, 52, 53].

To summarize, this study brings insights for the research field of natural nutrients in amelioration of the environmental hazards of toxic elements. In our holistic approach, we compared the effectiveness of two bee products with potential nutritional benefits. RJ appears to be more potent in mitigating the adverse effects of Cd. Therefore, further research is needed to help clarify the mechanism of action of RJ and complement the knowledge about its nutritional characteristics.

## Data Availability

All data generated and analyzed in this study are included in this article.
